# A reporter for noninvasively monitoring gene expression and plant transformation

**DOI:** 10.1038/s41438-020-00390-1

**Published:** 2020-09-19

**Authors:** Yubing He, Tao Zhang, Hui Sun, Huadong Zhan, Yunde Zhao

**Affiliations:** 1 State Key Laboratory of Crop Genetics and Germplasm Enhancement, Nanjing Agricultural University, Nanjing, 210095 China; 2 National Key Laboratory of Crop Genetic Improvement and National Center of Plant Gene Research (Wuhan), Huazhong Agricultural University, Wuhan, 430070 China; 3 Section of Cell and Developmental Biology, University of California San Diego, 9500 Gilman Drive, La Jolla, CA 92093-0116 USA; 4 Tata Institute for Genetics and Society-UCSD, La Jolla, CA 92093-0335 USA

**Keywords:** Plant biotechnology, Genetics

## Abstract

Reporters have been widely used to visualize gene expression, protein localization, and other cellular activities, but the commonly used reporters require special equipment, expensive chemicals, or invasive treatments. Here, we construct a new reporter *RUBY* that converts tyrosine to vividly red betalain, which is clearly visible to naked eyes without the need of using special equipment or chemical treatments. We show that *RUBY* can be used to noninvasively monitor gene expression in plants. Furthermore, we show that *RUBY* is an effective selection marker for transformation events in both rice and Arabidopsis. The new reporter will be especially useful for monitoring cellular activities in large crop plants such as a fruit tree under field conditions and for observing transformation and gene expression in tissue culture under sterile conditions.

## Introduction

Various genetically encodable reporters have been developed to monitor gene expression, protein subcellular localization, protein stability, hormonal signaling, and impacts of environmental signals. The green fluorescent protein (GFP) and its derivatives such as RFP, mCherry, and YFP have many applications as reporters for gene expression or as fusion proteins^1,2^. Although GFP is easy to use, it needs light sources to visualize the fluorescence signals. The β-glucuronidase (GUS) reporter has been widely used in plants for monitoring gene expression patterns and as a reporter for hormonal signaling^3^. For example, *DR5-GUS* transgenic lines are commonly used to monitor auxin distribution and auxin signaling^4^. Luciferase is another broadly used reporter in both animals and plants^5^. Both GUS and luciferase require the addition of expensive substrates X-Gluc (5-Bromo-4-chloro-1*H*-indol-3-yl β-d-glucopyranosiduronic acid) and luciferin, respectively. Whereas the traditional reporters have been very useful, they have limitations. Fluorescent proteins are often monitored under a microscope, rendering it less useful in analyzing plants in natural growing fields or analyzing large samples such as a tree. GUS-staining is invasive and often requires sacrifice of the plants. Luciferase can be used noninvasively, but it requires a special camera and spraying the expensive substrate. It is also not very practical to use them in fields. GUS and luciferase may not be optimal for sterile conditions such as tissue culture because addition of substrates increases the chance for contamination of microbes. Therefore, there is a need to develop new reporter systems that can be widely used to monitor cellular activities noninvasively, continuously, and cost-effectively. For the past few years, gene editing has been widely used in basic research and crop improvement. A visible marker for transgenes will greatly accelerate the isolation of edited plants that no longer harbor the gene editing machinery^6,7^.

**Fig. 1:**
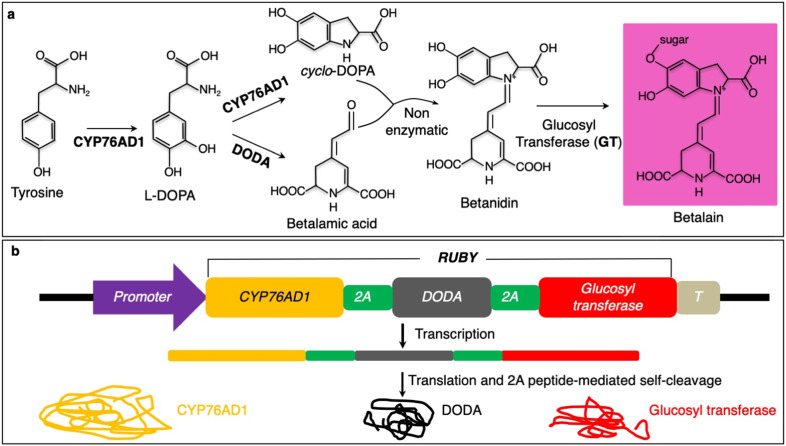
Components required for betalain biosynthesis. **a** Chemical reactions for converting tyrosine into betalain, which has a red color. Tyrosine is first oxidized by the P450 CYP76AD1 into l-3,4-dihydroxyphenylalanine (l-DOPA), which can be further converted into *cyclo*-DOPA by P450 CYP76AD1. In the presence of l-DOPA 4,5-dioxygenase (DODA), DOPA is oxidized and circularized into betalamic acid. *Cyclo*-DOPA condenses with betalamic acid, a non-enzymatic reaction, to produce betanidin. Glucoyslastion of betanidin generates the red color betalain. **b** A strategy for expressing the whole betalain biosynthetic pathway in a single cassette. The three betalain biosynthetic genes were fused into a single open reading frame, which can be expressed using a single promoter and terminator. Between the genes, sequences that encode 2A peptides were inserted. The 2A peptides undergo self-cleavage, thus releasing the individual enzymes for betalain biosynthesis. The betalain synthesis unit can be placed under the control of a promoter of interest. The terminator used here was the Arabidopsis *HSP18.2* terminator. The open reading frame of 2A-linked betalain biosynthesis genes is named *RUBY*.

Plants produce many colorful compounds that potentially can serve as reporters. For example, anthocyanins display bright red-blue colors and anthocyanin-producing rice plants have been used to generate interesting patterns in rice field. However, synthesis of anthocyanins requires multiple enzymes and varies greatly among different plants^8,9^. It is difficult to use anthocyanin biosynthesis pathways as a universal visible reporter. Betalains are a class of plant natural products derived from the amino-acid tyrosine^10,11^. The bright red color seen in beets, dragon fruit, Swiss chard, and other plants is resulted from accumulation of betalains. Biosynthesis of betalains has been well studied and only needs three enzymatic reactions to convert tyrosine into betalain (Fig. 1a)^12^. Tyrosine is first hydroxylated on the benzene ring, resulting in l-3,4-dihydroxyphenylalanine (l-DOPA) (Fig. 1a). The reaction is catalyzed by the P450 oxygenase CYP76AD1 (Fig. 1a). l-DOPA can be further oxidized into *cyclo*-DOPA by CYP76AD1 (Fig. 1a). Alternatively, l-DOPA is catalyzed by l-DOPA 4,5-dioxygenase (DODA) into betalamic acid, which is subsequently condensed with *cyclo*-DOPA into betanidin. The condensation reaction does not require an enzyme (Fig. 1a). Finally, a sugar moiety is added to betanidin by a glucosyltransferase to generate the colorful betalain (Fig. 1a). Betalain has a very bright red color, which potentially can serve as a reporter to track gene expression or to visualize transgenic events. Because every cell contains the amino-acid tyrosine, exogenous application of tyrosine to tissues may not be required. We hypothesized that betalain would be a more convenient reporter than the aforementioned reporters. It is visible to naked eyes without any needs for special equipment. It does not require processing samples and it allows continuously monitoring events throughout the life cycle of an organism. Moreover, it is applicable to large plants grown under normal field conditions. Herein, we synthesize an artificial open reading frame named *RUBY* that when expressed can produce all of the enzymes required for betalain biosynthesis. We show that *RUBY* is a very effective marker for noninvasively selecting transformation events in both rice and Arabidopsis. Moreover, we show that *RUBY* can be used to visualize gene expression without any chemical treatments or special equipment, providing useful tools for visualizing gene expression in large plants under natural field growth conditions.

## Results and discussion

### Construction of a single open reading frame for the betalain biosynthetic pathway

Heterologous expression of *CYP76AD1*, *DODA* in tobacco, and other plants demonstrated that the betalain biosynthetic pathway can be re-constituted in plant cells^13,14^. In order to use betalain as a visual reporter, we need to effectively co-express the entire pathway using a single promoter. We organized *CYP76AD1*, *DODA*, and *Glucosyltransferase* into a single open reading frame (Fig. 1b) (Supplemental Fig. 1). The stop codons of *CYP76AD1* and *DODA* were removed. The three genes were linked by sequences that encode 2A peptides (Fig. 1b) (Supplemental Fig. 1)^15,16^. Upon transcription, the single transcript, which includes the coding regions of the three enzymes, produced the three separate enzymes through either 2A-mediated self-cleavage or ribosomal “skipping” (Fig. 1b)^17^. The 2A system enables the expression of multiple proteins under the control of a single promoter. We name the 2A-linked unit of *CYP76AD1*, *DODA*, and *Glucosyltransferase RUBY* (Fig. 1b). *RUBY* can be expressed when a promoter is placed in front of it. The expression pattern and level of a particular gene may be inferred from the red color of betalain if the gene’s promoter is used to drive *RUBY* expression.

### *RUBY* is capable of synthesizing betalain in tobacco

We first placed *RUBY* under the control of Cauliflower Mosaic Virus (CaMV) *35S* promoter, which is a widely used constitutively strong promoter^18^. To test whether *RUBY* can produce functional enzymes for betalain synthesis, we infiltrated tobacco leaves with Agrobacteria that contain *RUBY*-expressing plasmid (Supplemental Fig. 2). Transient expression of *RUBY* led to the production of betalain in tobacco leaves, suggesting that the synthetic open reading frame *RUBY* can produce the functional enzymes for the synthesis of betalain. Moreover, we observed that betalain was not transported from the spots of *Agrobacterium*-infiltration spots to other leaves of the plant (Supplemental Fig. 2).

### Synthesis of betalain by *RUBY* in *Arabidopsis*

We transformed the *35S:RUBY* construct into Arabidopsis using *Agrobacterium*-mediated floral dipping^19^. Two days after floral dipping, we noticed that the transformed plants displayed patches of red color (Supplemental Fig. 3a), indicating that the *RUBY* cassette was functionally expressed and that *RUBY* may be used to monitor transient *Arabidopsis* transformation. Once the seeds from the *Agrobacterium*-dipped plants were harvested, transgenic seeds could be easily differentiated from non-transgenic seeds (Supplemental Fig. 3b). The transformed seeds had a dark red color (Supplemental Fig. 3b), demonstrating that *RUBY* can be used as a visual selection marker for transgenic events in Arabidopsis. We previously used mCherry as a very effective marker to select transgenic events (Gao et al., 2016), which requires a dissecting microscope with fluoresence capability. *RUBY* is a better option because it does not require special equipment.

**Fig. 2:**
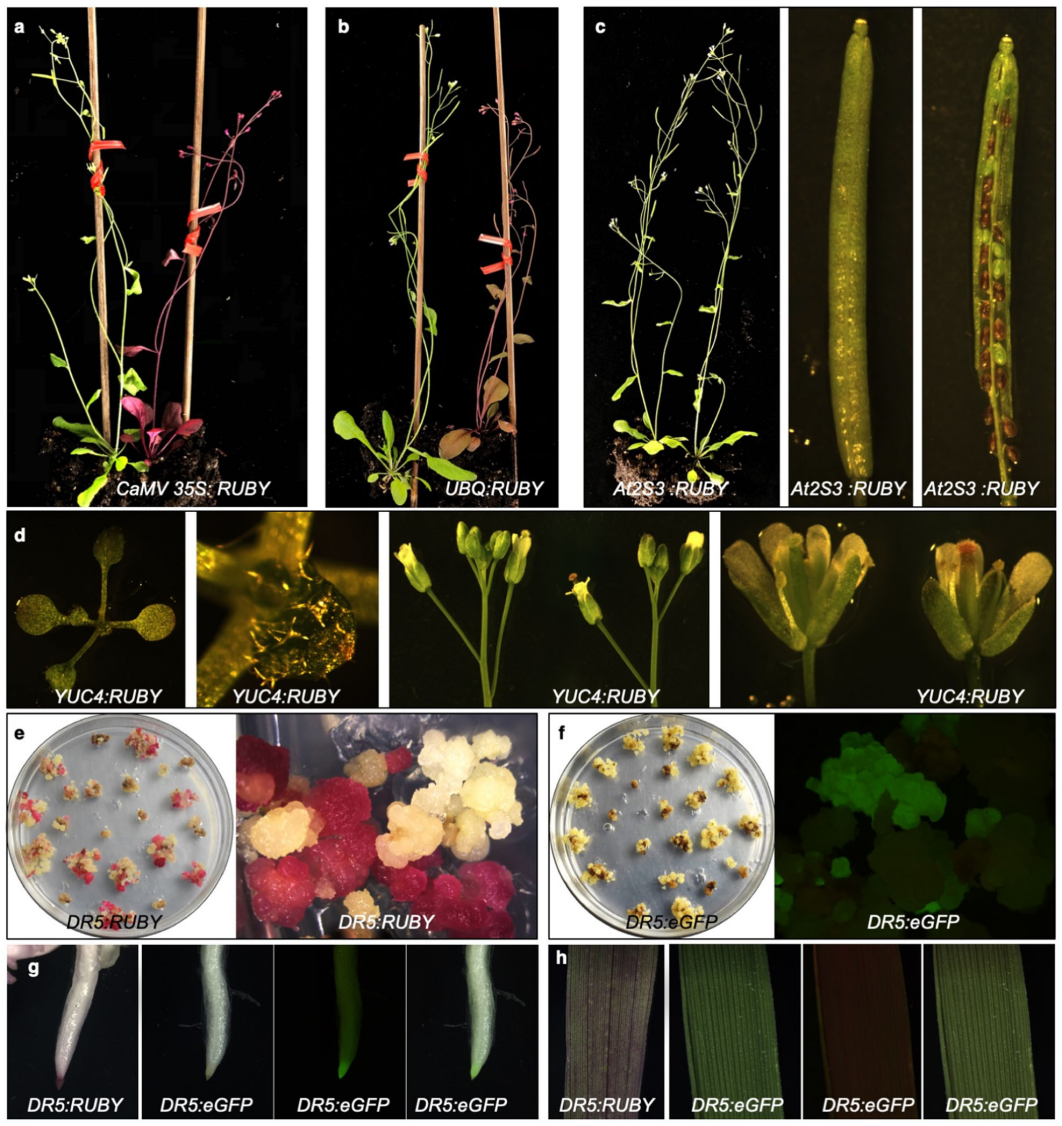
RUBY serves as an effective reporter for gene expression and plant transformation. **a** *RUBY* expression driven by the *CaMV 35S* promoter led to a red Arabidopsis plant (right) compared with the WT plant (left). **b** *UBIQUITIN* promoter was effective in driving *RUBY* expression throughout the plant (right). Non-transgenic WT was shown left. **c** The seed specific promoter *At2S3* did not lead to *RUBY* expression in leaves and stems. The transgenic plant (right) and non-transgenic plant (left) were indistinguishable. The siliques of *At2S3:RUBY* were similar to those of WT, however, when the silique was opened, the seeds of *At2S3:RUBY* were clearly red. Moreover, it was obvious that transgenic and no-transgenic seeds were segregating in a silique from a T1 *At2S3:RUBY* plant. **d** *YUC4:RUBY* plants displayed patches of red at the tip of leaves and apical region of a gynoecium. **e** *DR5:RUBY* was expressed in rice calli. The red color can be used to distinguish transgenic (red) and non-transgenic calli (white). *DR5:eGFP* has been used in rice calli **f**, but it was much more difficult to distinguish transgenic from non-transgenic using *DR5:eGFP* compared with *RUBY*. **g** Roots of *DR5:RUBY and DR5eGFP* rice plants, which had similar patterns. The three *DR5:eGFP* pictures were generated with the same root: bright field (left), 488 nm fluorescence field (middle), and the merged (right). **h** Activation of DR5 promoter in *DR5:RUBY* leaves was easy to observe whereas *DR5:eGFP* was much more difficult to detect: bright field (left), 488 nm fluorescence field (middle), and the merged (right).

The *35S:RUBY* plants produced sufficient amount of betalain to become visually evident (Fig. 2a). Consistent with previous reports that *CaMV 35S* promoter is constitutively active, we observed red color in all tissues throughout the plant life cycle (Fig. 2a) (Supplemental Fig. 3c, d). We also expressed *RUBY* reporter under the control of the Maize *UBIQUITIN* promoter, which has been widely used to overexpress genes in monocots^20^. Similar to *35S:RUBY* plants, *UBQ:RUBY* plants were also visibly red in leaves, stem, and flowers (Fig. 2b). These results clearly demonstrated that *RUBY* could be expressed in *Arabidopsis* and that our *RUBY* reporter was able to functionally re-constitute the betalain biosynthetic pathway.

We expressed *RUBY* using the seed specific *At2S3* promoter, which we previously used to drive *mCherry* expression in Arabidopsis to facilitate the selection of transgenes^6^. As shown in Fig. 2c, the transgenic plants were indistinguishable from wild type plants. When we checked the seeds in a silique from an *At2S3:RUBY* T1 plant, *RUBY*-expressing seeds displayed strong red color, whereas the non-transgenic seeds were green (Fig. 2c). *RUBY* can be conveniently used to select single T-DNA insertion events by analyzing the ratio of red seeds to green seeds, which should be ~3:1 for single insertions. The *At2S3:RUBY* results demonstrate that *RUBY* could be an effective marker for Arabidopsis transformation. Furthermore, betalain was not widely transported from the sites of synthesis to other tissues as we did not see any red color in leaves (Fig. 2c). We also expressed *RUBY* under the control of the Arabidopsis *YUC4* promoter (Fig. 2d). *YUC4*, which encodes a key enzyme in auxin biosynthesis, was shown to express in small regions of embryos, leaves, and flowers^21,22^. GUS signals were observed in leaf tips and apical region of a gynoecium in *YUC4 promoter:GUS* transgenic plants. We observed similar patterns of betalain production in *YUC4:RUBY* lines (Fig. 2d).

### *RUBY* synthesizes betalain in rice

Unlike *Arabidopsis*, rice and many other plants are transformed through tissue culture and the formation of calli, which are often mosaic. A visible marker for transformation at tissue culture stage will be very useful. We placed *RUBY* under the control of *DR5*, an auxin-responsive synthetic promoter^4^, which was used to monitor stem cell initiation during tissue culture. We also used the rice *ACTIN1* promoter, which is considered a strong promoter, to control *RUBY* expression. We transformed the *DR5:RUBY* (Fig. 2e) and *OsACTIN1: RUBY* (Supplemental Fig. 4) into rice calli. *RUBY* expression rendered calli vividly red. The presence of the *RUBY* gene corelated to the observed *RUBY* expression in rice calli (Supplemental Fig. 4). We also observed a positive correlation between the brightness of the calli the expression levels of *RUBY* gene (Supplemental Fig. 4). Introduction of *RUBY* made it easier to distinguish the transformed calli from untransformed calli (Fig. 2e). *RUBY* enables selection of calli that have better expression of transgenes among the hygromycin-resistant calli in the same tissue block (Supplemental Fig. 4). In comparison, *DR5:eGFP* has been used to visualize transgenic calli (Fig. 2f). Our *RUBY* system is clearer and much more convenient to operate during tissue culture condition. The *DR5:RUBY* and *DR5:eGFP* in rice roots showed similar patterns (Fig. 2g), but *DR5:RUBY* was easier to observe without any treatments or a change in light conditions. The advantages of *DR5:RUBY* over *DR5:eGFP* was also obvious in rice leaves (Fig. 2h). Moreover, *RUBY* as a visible marker was very useful for monitoring transgenes in intact adult rice plants (Supplemental Fig. 5).

We demonstrate that our synthetic cassette of betalain biosynthetic genes was able to produce betalain in *Arabidopsis* and in rice, providing a visible color for monitoring gene expression and plant transformation. We believe that *RUBY* will be very useful in large plants such as fruit trees and in field conditions. Because *RUBY* does not require either special equipment or expensive substrates, *RUBY* provides a cost-effective reporter and *RUBY* is a convenient alternative to the existing reporters. We envision that *RUBY* can be adapted for applications in some microbes and animals because the substrate tyrosine exists in all cells. For example, *RUBY* may provide a more convenient marker than β-galactosidase (LacZ) in yeast two-hybrid screens. Betalain is a natural product and was shown to have health benefits. Using *RUBY* as a reporter has less environmental and health concerns compared with antibiotic and/or herbicide resistance markers.

## Materials and methods

### *Arabidopsis* constructs and transformation

The backbone of the *RUBY* constructs for Arabidopsis is the plasmid *pHDE*, which was described previously^6^. We took advantage of the P2A peptide, which has the sequence of (GSGATNFSLLKQAGDVEENPGP), to link the *CYP76AD1*, *DODA*, and *glucosyltransferase* (*GT*) coding regions. The transcriptional terminator used was the *HSP18.2* terminator from Arabidopsis. The 2A-linked *CYP76AD1*, *DODA*, and *GT* unit was named *RUBY*, which was cloned into *pHDE* along with the HSP terminator by Gibson assembly at the *Xba*I^23^. The entire sequence of *RUBY* and terminator was shown in Supplemental Fig. 1.

Various promoters can be cloned into the *Pme*I site of *pHDE-RUBY* to drive *RUBY* expression. Promoters were amplified by PCR and the primers used in this study were listed in Supplemental Table 1. We expressed *RUBY* using *CaMV 35S*, Maize *UBIQUITIN* promoter, *At2S3* promoter, and *YUC4* promoter to test whether *RUBY* can serve as an effective reporter. The constructs were transformed into Arabidopsis Columbia plants by *Agrobacterium-*mediated floral dipping^19^. Transgenic seeds for *35S:RUBY*, *UBQ:RUBY*, and *At2S3: RUBY* were easily identified by red color. Transgenic plants for *YUC4:RUBY* were selected on MS medium containing hygromycin (16.7 μg/ml).

### Rice constructs and transformation

The auxin-responsive promoter (*DR5*) was composed of a synthetic promoter with 16 repeats of core auxin response element (AuxRE) sequence (TGTCTC) linked with CaMV minimal *35S* promoter^4^. The *DR5* promoter was synthesized and then cloned into *pDX2181*^24^ between *BamH* I and *Pst* I sites by Gibson assembly with primer pair Oligo15 and Oligo16 (Supplemental Table 1), resulting in the plasmid *pDR5:eGFP*.

To construct the *pDR5:RUBY* plasmid, we linked the three betalain biosynthetic genes through 2A peptides. Here, we used the F2A peptide, which has the following peptide sequence: QLLNFDLLKLAGDVESNPGP. The *RUBY* cassette replaced the *eGFP* in the *pDR5:eGFP* plasmid, resulting in the *pDR5:RUBY*. The rice version of *RUBY* was also detailed in the Supplemental Fig. 1. Both the *DR5:RUBY* and *DR5:eGFP* plasmids were transformed into Xiaowei^NIP^^25^ through *Agrobacterium*-mediated plant transformation following a protocol that was previously described^26^.

### Quantitative analysis the expression level of *RUBY*

The relative expression levels of *RUBY* in the transgenic calli of *OsACTIN1: RUBY* were determined by reverse transcription quantitative PCR using the primer pair Oligo26/Oligo27. The primer pair Oligo28/ Oligo29 was specific for the rice *UBIQUITIN* (*UBQ*) gene, which served as the endogenous reference gene^27^. The primers were listed in Supplemental Table 1.

## Supplementary information

Supplemental figures and Table

## Data Availability

The plasmids will be available at Addgene.
