# Supplementary material for: A reporter for noninvasively monitoring gene expression and plant transformation
Source: Hortic Res. 2020 Sep 19;7:152. doi: 10.1038/s41438-020-00390-1 (PMC7502077; doi:10.1038/s41438-020-00390-1)
Supplement: Supplementary file 1 — Supplemental figures and Table [file 41438_2020_390_MOESM1_ESM.pdf]

ATGGATCATGCGACCCTCGCCATGATCCTCGCGATCTGGTTCATCAGCTTCCACTTCATCAAGC
TGCTGTTCTCCCAGCAGACCACCAAGCTGCTTCCGCCAGGACCAAAGCCGCTTCCGATCATCGG
CAACATCCTTGAGGTGGGCAAGAAGCCGCATCGGTCCTTCGCCAACCTCGCCAAGATTCACGGC
CCACTCATTTCCCTCAGACTCGGCTCTGTGACCACCATCGTTGTGTCCTCTGCCGACGTGGCCA
AAGAGATGTTCCTCAAGAAGGATCACCCGCTCTCCAACCGCACGATCCCGAATAGTGTTACAGC
CGGCGACCACCACAAGCTCACCATGTCTTGGCTCCCGGTGTCTCCGAAGTGGCGCAACTTCCGC
AAGATTACCGCCGTGCATCTGCTCTCCCCACAGAGACTCGATGCCTGCCAGACATTCAGGCACG
CCAAGGTGCAGCAGCTCTACGAGTACGTTCAAGAGTGCGCCCAGAAAGGCCAGGCCGTGGATAT
TGGCAAGGCCGCCTTTACGACCAGCCTCAACCTCCTCAGCAAGCTGTTCTTCAGCGTCGAGCTG
GCGCACCACAAGTCCCATACCAGCCAAGAGTTCAAAGAGCTGATCTGGAACATCATGGAAGATA
TAGGCAAGCCGAACTACGCCGACTACTTCCCGATTCTCGGCTGCGTTGACCCATCTGGCATTAG
AAGAAGGCTCGCCTGCTCCTTCGACAAGCTGATCGCCGTGTTCCAGGGCATCATCTGCGAGAGA
CTCGCCCCAGATTCCTCCACCACAACTACCACCACCACCGACGACGTGCTCGATGTGCTCCTCC
AGCTGTTCAAGCAGAACGAGCTGACGATGGGCGAGATCAACCACCTCCTCGTGGACATCTTCGA
CGCCGGCACCGATACCACATCCTCCACATTCGAGTGGGTGATGACCGAGCTGATCCGCAATCCA
GAGATGATGGAAAAGGCCCAAGAGGAAATCAAGCAGGTCCTCGGCAAGGACAAGCAGATCCAAG
AGTCCGACATCATCAACCTGCCGTACCTCCAGGCGATCATCAAAGAGACACTCCGCCTCCATCC
GCCGACCGTGTTCTTGCTCCCAAGAAAGGCCGACACCGATGTCGAGCTGTACGGCTACATCGTG
CCGAAGGATGCCCAGATCCTCGTGAACCTCTGGGCCATTGGCAGGGACCCAAACGCCTGGCAGA
ACGCCGATATTTTCAGCCCAGAGCGCTTCATCGGCTGCGAGATCGATGTTAAGGGCCGCGATTT
CGGCCTCCTTCCATTTGGCGCTGGCCGCAGAATTTGCCCAGGCATGAATCTCGCCATCAGGATG
CTCACCCTCATGCTCGCCACACTCCTCCAGTTCTTCAACTGGAAGCTCGAAGGCGACATCTCCC
CGAAGGACCTCGACATGGACGAGAAGTTCGGCATTGCGCTCCAAAAGACCAAGCCGCTCAAGCT
CATCCCGATTCCGCGCTAC**GGAAGCGGAGCTACTAACTTCAGCCTGCTGAAGCAGGCTGGAGAC**
GTGGAGGAGAACCCTGGACCTATGAAGATGATGAACGGCGAGGACGCCAACGACCAGATGATCA
AAGAGTCCTTCTTCATCACCCACGGCAACCCGATCCTCACCGTCGAGGATACACATCCGCTCAG
GCCGTTCTTCGAGACATGGCGCGAGAAGATTTTCTCCAAGAAGCCGAAGGCCATCCTCATCATC
TCCGGCCACTGGGAGACAGTGAAGCCAACCGTGAACGCCGTGCACATCAACGACACCATCCACG
ACTTCGACGACTACCCAGCCGCCATGTACCAGTTCAAGTACCCAGCTCCAGGCGAGCCAGAGCT
TGCGAGAAAGGTGGAAGAGATCCTCAAGAAGTCCGGGTTCGAGACAGCCGAGACAGACCAAAAG
AGGGGCCTTGATCACGGCGCCTGGGTTCCACTCATGCTCATGTATCCAGAGGCGGACATCCCGG
TGTGCCAGCTCTCAGTTCAGCCACATCTCGACGGCACCTACCACTACAATCTCGGCAGAGCCCT
CGCGCCGCTCAAGAATGATGGCGTGCTCATTATTGGCTCCGGCAGCGCCACACATCCACTCGAT
GAGACACCGCACTACTTCGATGGTGTTGCCCCTTGGGCCGCTGCCTTCGATTCTTGGCTTAGGA
AGGCCCTCATCAACGGCCGCTTCGAGGAAGTGAACATCTACGAGAGCAAGGCCCCGAACTGGAA
GCTCGCCCATCCATTTCCAGAGCACTTCTACCCGCTCCACGTTGTGCTCGGCGCTGCTGGTGAA
AAGTGGAAGGCCGAGCTGATCCACTCCTCCTGGGATCATGGCACACTTTGCCACGGCTCCTACA
AGTTCACCTCCGCC**GGTTCAGGTGCCACTAATTTTTCTCTCTTGAAACAGGCCGGTGACGTTGA**

AGAGAACCCAGGTCCAATGACCGCCATCAAGATGAACACCAACGGCGAGGGCGAGACACAGCAC
ATCCTCATGATCCCGTTCATGGCGCAGGGCCACCTCAGGCCATTTCTCGAACTCGCCATGTTCC
TCTACAAGCGCTCCCACGTGATCATCACCCTGCTCACAACTCCGCTCAACGCCGGCTTCCTCAG
GCACCTCCTTCACCACCATTCCTACTCCTCCAGCGGCATCAGGATCGTCGAGCTGCCATTCAAC
TCCACCAACCACGGACTCCCACCGGGCATCGAGAACACCGATAAGCTCACACTCCCGCTCGTGG
TGTCCCTCTTCCATTCCACCATCAGCCTCGATCCGCACCTCCGCGATTACATCTCCAGGCATTT
CAGCCCAGCCAGGCCACCACTCTGCGTGATCCATGATGTGTTCCTCGGCTGGGTTGACCAGGTG
GCCAAGGATGTGGGCTCTACAGGCGTGGTGTTCACAACAGGCGGCGCTTATGGCACATCCGCCT
ACGTGTCCATCTGGAACGATCTCCCGCACCAGAACTACTCCGACGACCAAGAGTTCCCGCTGCC
AGGCTTCCCAGAGAACCATAAGTTCCGCAGGTCCCAGCTCCATCGGTTCCTCAGATATGCCGAC
GGCTCCGACGATTGGTCCAAGTATTTCCAGCCGCAGCTCCGCCAGTCCATGAAGTCTTTTGGCT
GGCTCTGCAACTCCGTGGAAGAGATCGAGACACTCGGCTTCTCCATCCTCCGCAACTACACCAA
GCTGCCGATCTGGGGCATCGGCCCACTTATTGCTTCCCCAGTGCAGCACTCCTCCTCCGACAAC
AATTCAACAGGCGCCGAGTTCGTGCAGTGGCTCAGCCTCAAAGAGCCGGACTCCGTCCTCTACA
TCTCCTTCGGCTCCCAGAACACGATCAGCCCGACGCAGATGATGGAACTCGCTGCTGGCCTTGA
GTCCTCCGAGAAGCCATTCCTCTGGGTGATCAGAGCCCCGTTCGGCTTCGACATCAACGAAGAG
ATGCGCCCAGAGTGGCTGCCAGAGGGCTTTGAGGAACGCATGAAGGTGAAGAAACAGGGCAAGC
TCGTGTACAAGCTCGGCCCGCAGCTTGAGATCCTCAACCATGAATCCATCGGCGGCTTTCTCAC
CCACTGCGGATGGAACAGCATCCTTGAGTCTCTTCGCGAGGGCGTTCCGATGCTTGGATGGCCA
CTTGCTGCCGAGCAGGCCTACAACCTCAAGTACCTCGAAGATGAGATGGGCGTCGCGGTTGAGC
TTGCTAGAGGCCTCGAAGGCGAGATCTCCAAAGAGAAGGTCAAGCGCATCGTCGAGATGATCCT
TGAGCGCAACGAGGGCTCCAAAGGCTGGGAGATGAAGAATCGCGCCGTGGAAATGGGCAAAAAG
CTCAAGGACGCCGTGAACGAGGAAAAAGAGCTGAAGGGCTCCTCCGTGAAGGCGATCGACGATT
TCCTCGACGCCGTCATGCAGGCCAAACTTGAGCCAAGCCTCCAGTGATAGTGA
ATATGAAGATGAAGATGAAATATTTGGTGTGTCAAATAAAAAGGTTGTGTGCTTAAGTTTGTGT
TTTTTTCTTGGCTTGTTGTGTTATGAATTTGTGGCTTTTTCTAATATTAAATGAATGTAACATC
TCATTATAATGAATAAACAAATGTTTCTATAATCCATTGTGAATGTTTTGTTGGATCTCTTCTC
CAGCATATAACTACTGTATGTGCTATGGTATGGACTATGGAATATGATTAAAGATAAG

Supplemental Fig. 1. The complete sequence of *RUBY* reporter. The betalain biosynthetic genes *CYP76AD1* (highlighted in light blue), *DODA* (yellow), and *Glucosyltransferase* (gray), were arranged as a single open reading frame. The three genes were linked by sequences (red) that encode 2A peptides. Note that the two 2A sequences differed in DNA sequences, but both encoded the same peptide, which had a sequence of GSGATNFSLLKQAGDVEENPGP. The RUBY ORF was followed by the Arabidopsis *HSP18.2* terminator. For rice plasmids, we used F2A peptide (QLLNFDLLKLAGDVESNPGP). The corresponding DNA sequence is: CAATTGTTGAATTTTGATTTGTTGAAGTTGGCTGGAGATGTTGAATCTAATCCTGGAC CT

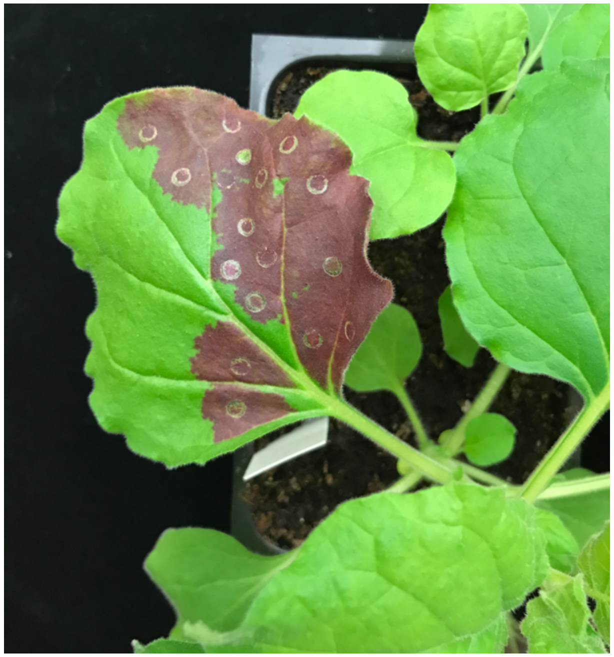

Supplemental Fig. 2. Transient expression of *RUBY* in tobacco leaves.
Agrobacteria that contain the expression cassette of *RUBY* under the control of *CaMV 35S* promoter were infiltrated into tobacco leaves. The picture was taken 72 hours after infiltration.

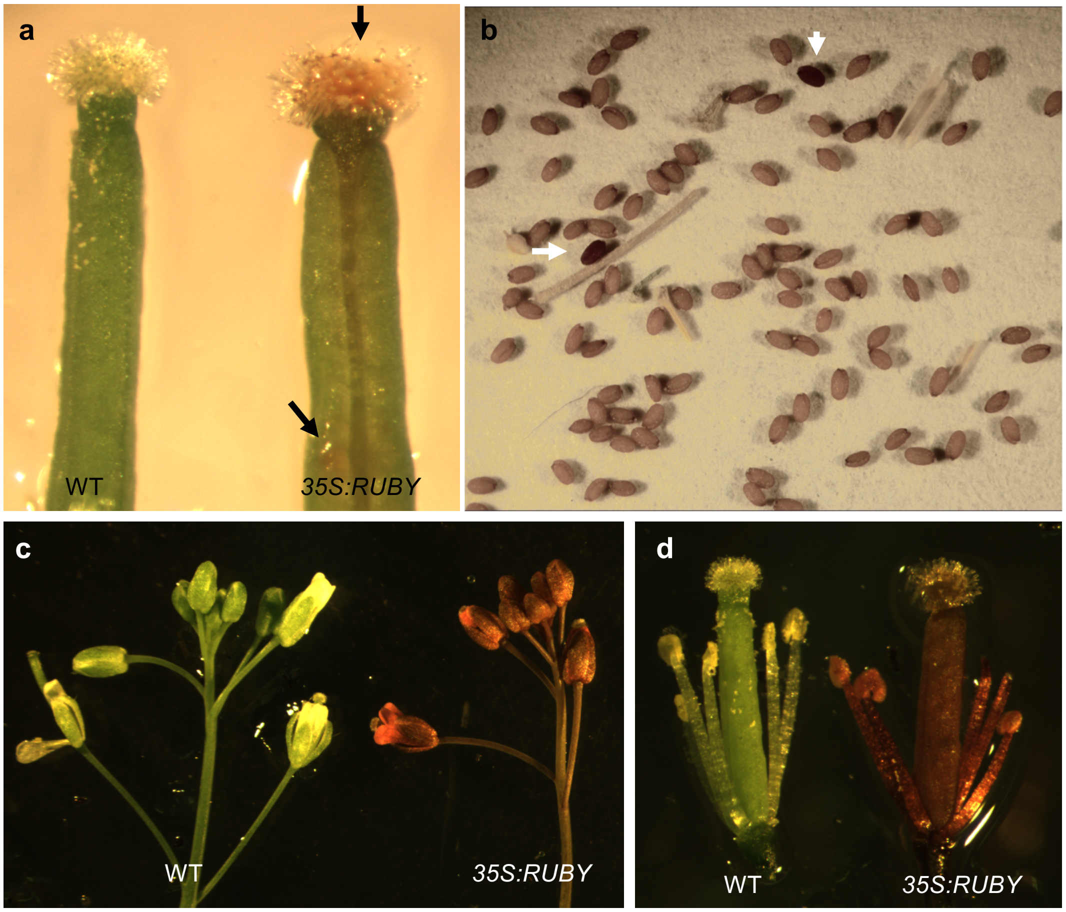

Supplemental Fig. 3. Expression of *RUBY* driven by the *CaMV 35S* promoter in Arabidopsis. **a**) Two days after floral dipping, red betalain was visible in some flowers (arrow) (right), suggesting that the betalain enzymes were produced transiently. **b***)* Transgenic seeds (arrow) were easily distinguishable from non-transgenic seeds among the seeds harvested from plants underwent floral dipping. **c**)T1 plants of *35S:RUBY* (right) had red flowers and red stem . **d)** Betalain was synthesized in all floral organs in *35S:RUBY* plants.

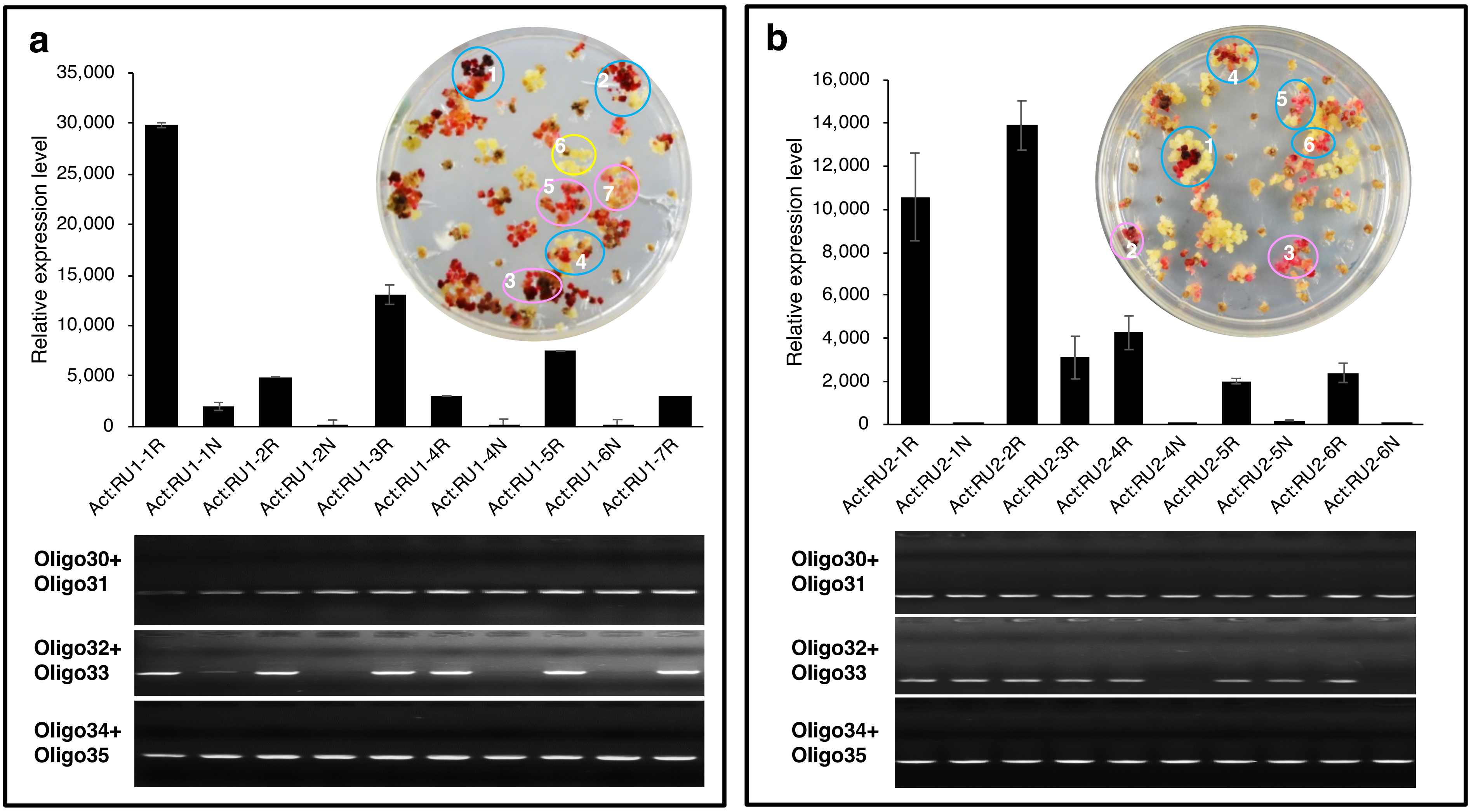

Supplemental Fig. 4. Correlation of *RUBY* expression and the brightness of betalain color. Independent hygromycin-resistant calli tissue blocks were used for quantitative analysis of the expression level of *RUBY* from two independent *OsACTIN1: RUBY* transformation experiments **(a)** and **(b)**. *Act: RU1* and *Act: RU2* refer to plate 1 and plate 2 of two independent *OsACTIN1: RUBY* transformation events. The calli samples used for amplifying the DNA fragments of *RUBY* and *HptII,* and analyzing the *RUBY* expression level were numbered and labeled by circles with different colors. The circles in pink and yellow represent only red (R) and yellow (N) calli, respectively. The blue circle refers to both the red (R) and yellow (N) calli in same tissue block were analyzed separately. “R” in Act:RU1-1R and other terms refer to red tissue while “N” in the terms refer to negative of RUBY phenotypes. Oligo30/Oligo31, Oligo32/Oligo33 and Oligo34/Oligo35 refer the primer pairs used for detecting the presence the rice genome DNA, *RUBY* DNA and selection marker gene *HptII*, respectively. The primers were described in Supplemental Table 1.

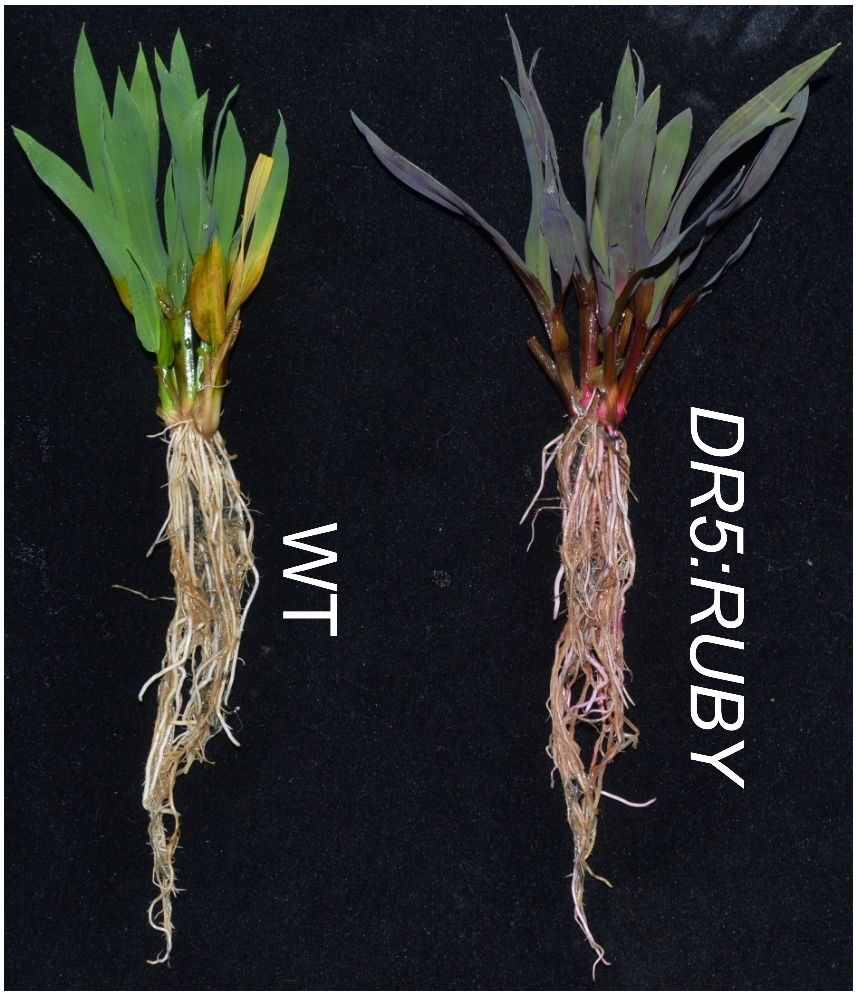

Supplemental Fig. 5. RUBY is useful for monitoring transgenes in intact rice plants. The expression of *DR5:RUBY* in Xiaowei^{NIP} background was obvious in one month old plant grown in the field. WT refers to Xiaowei^{NIP} plant.

Primer name	Sequence	Annotation
**Oligo1**	CACTGATAGTTTAAACTAGTATGGATCATGCGACCCTCGC	*RUBY* construction
**Oligo2**	CACCTGCCTGCTTAAGGAGGCTAAAATTGGTAGCTCCGCTACCGTAGCGCGGAATCGGGA	*RUBY* construction
**Oligo3**	CCTTAAGCAGGCAGGTGATGTAGAAGAGAACCCCGGGCCTATGAAGATGATGAACGGCGA	*RUBY* construction
**Oligo4**	CTCCTGCTTGCTTGAGCAGGCTAAAGTTGGTTGCTCCGGATCCGGCGGAGGTGAACTTGT	*RUBY* construction
**Oligo5**	GCTCAAGCAAGCAGGAGATGTTGAGGAAAATCCTGGCCCCATGACCGCCATCAAGATGAA	*RUBY* construction
**Oligo6**	GCTAGCTTACTCAGTTAGGTCTTATCTTTAATCATATTCC	*RUBY* construction
**Oligo7**	CTGTCAAACACTGATAGTTTTGAGACTTTTCAACAAAGGG	*35S:RUBY* construction
**Oligo8**	CGCATGATCCATACTAGTTTTCAGCGTGTCCTCTCCAAAT	*35S:RUBY* construction
**Oligo9**	CTGTCAAACACTGATAGTTTCTGCAGTGCAGCGTGACCCG	*ZmUbq:RUBY* construction
**Oligo10**	CGCATGATCCATACTAGTTTCTGCAGAAGTAACACCAAAC	*ZmUbq:RUBY* construction
**Oligo11**	CTGTCAAACACTGATAGTTTATGTCCAACATGCATGCG	*AtYUC4:RUBY* construction
**Oligo12**	CGCATGATCCATACTAGTTTGTCGACTAATAAAAGCGAAAGAGAG	*AtYUC4:RUBY* construction
**Oligo13**	CTGTCAAACACTGATAGTTTTAAGCTGGCACAACTATATT	*AtAT2S3:RUBY* construction
**Oligo14**	CGCATGATCCATACTAGTTTTGTTGGTACCGTTTTGCTAT	*AtAT2S3:RUBY* construction
**Oligo15**	TAAGGGACTGACCACCCGGGGATCCGGTATCGATAAGCTTGCAGCC	*pDR5:eGFP* construction
**Oligo16**	CAGCGCTGAAGCTTGGCTGCAGTGTAATTGTAAATAGTAATTGTAATG	*pDR5:eGFP* construction
**Oligo17**	CACTGCAGCCAAGCTTCAGCGCTGTAGATCTCCATGGATCATGCGACCCTCG	*pDR5:RUBY* construction
**Oligo18**	CATCTCCAGCCAACTTCAACAAATCAAAATTCAACAATTGGTAGCGCGGAATCGGGATG	*pDR5:RUBY* construction
**Oligo19**	GTTGAAGTTGGCTGGAGATGTTGAATCTAATCCTGGACCTAAGATGATGAACGGCGAGG	*pDR5:RUBY* construction
**Oligo20**	CGATCGGGGAAATTCGAGCTGGTCACCGGCGGAGGTGAACTTGTAGG	*pDR5:RUBY* construction
**Oligo21**	GTTGAAGTTGGCTGGAGATGTTGAATCTAATCCTGGACCTACCGCCATCAAGATGAACA	*pDR5:RUBY* construction
**Oligo22**	TCTTCTAACATGCGGTGACGTGGAGGAGAATCCCGGCCCTACCGCCATCAAGATGAACA	*pDR5:RUBY* construction
**Oligo23**	ACGATCGGGGAAATTCGAGCTGGTCACCTCACTATCACTGGAGGCTTG	*pDR5:RUBY* construction
**Oligo24**	CCTACAAGTTCACCTCCGCCGAGGGCAGAGGAAGTCTTCTAACATGCGGTGACGTGGAG	*pDR5:RUBY* construction
**Oligo25**	CTACAAGTTCACCTCCGCCCAATTGTTGAATTTTGATTTGTTGAAGTTGGCTGGAGATG	*pDR5:RUBY* construction
**Oligo26**	AACAGCATCCTTGAGTCTCTTCG	qPCR primer of *RUBY*
**Oligo27**	TTCTCTTTGGAGATCTCGCCTTC	qPCR primer of *RUBY*
**Oligo28**	AACCAGCTGAGGCCCAAGA	qPCR primer of endogenous reference gene
**Oligo29**	ACGATTGATTTAACCAGTCCATGA	qPCR primer of endogenous reference gene
**Oligo30**	CTCAACCCCAAGGCTAACAG	detection the presence of rice genome DNA
**Oligo31**	ACCTCAGGGCATCGGAAC	detection the presence of rice genome DNA
**Oligo32**	AGAAGATTTTCTCCAAGAAGCCGA	detection the presence of *RUBY* DNA
**Oligo33**	ATAATGAGCACGCCATCATTCTTG	detection the presence of *RUBY* DNA
**Oligo34**	ACGGTGTCGTCCATCACAGTTTGCC	detection the presence of *HptII* gene
**Oligo35**	GGAGAGGACACGCTGAAATCACCA	detection the presence of *HptII* gene

Supplemental Table I: Primers used in this study.
